# Twilight Sleep

**Published:** 1920-03-06

**Authors:** 


### Twilight Sleep.

Any proposals which may influence the question of birth-rate necessarily concern the authorities, and among those which the annual official health review considers is a method of conducting confinement so that all recollection of its incidents is lost, there being apparently no record in the memory of their occurrence. This is known as the "Scopolamine Morphine Narcosis," or, in popular terms, "Twilight Sleep." Scopolamine is an alkaloid, present in henbane, in belladonna, and other plants, which has the property of preventing the physiological recording of sensations. A committee of the Section of Obstetrics and Gynæcology of the Royal Society of Medicine was formed to study its action, and has reported. Observations were recorded in the lying-in wards of St. Thomas's and St. Bartholomew's Hospitals, at Queen Charlotte's Lying-in Hospital, the City of London Lying-in Hospital, and the General Lying-in Hospital, York Road.

The results showed (*a*) that if the treatment were carried out carefully, and with all precautions that experience indicated, risk to the life of the mother was not increased, but that there was some added risk to the life of the child; (*b*) that the object of the treatment—*i.e.*, absence of recollection of labour and its incidents, was effected in a large proportion of cases; (*c*) that the treatment required a nurse or midwife to be constantly present with the patient; (*d*) that a medical practitioner should see her at frequent intervals; and (*e*) that it could not be carried out at the patient's home except for persons to whom the cost was immaterial. The report says it would therefore seem that the wider adoption of this treatment must for the present be limited to hospitals. Such hospitals would require a greatly increased nursing staff, and a resident medical attendant, if they undertook the treatment. A considerable proportion of the lying-in hospitals and homes at present assisted by the Board's grant have no resident medical attendant.

Several months ago in this Journal (Feb. 22, 1919, p. 443) we gave a complete description of this method as it might be expected to work if adopted throughout the length of the country. True, there is still some difference of opinion among members of the profession as to the advisability of such a step, but the bulk of those in favour undoubtedly consist of those specially trained in actual use of the method. Two facts make to our mind, particularly at the present time, a strong contra-indication. From the medical attendant a highly specialised knowledge is required, if either mother or child are not to run grave risk, and the child's prospects in cases with even a low degree of obstructed labour are far from good even when institutional figures alone are consulted. The method is quite unsuited to the average middle or lower-class house.

